# Giant Arachnoid Granulations: Diagnostic Workup and Characterization in Three Symptomatic Adults

**DOI:** 10.3390/ijms241411410

**Published:** 2023-07-13

**Authors:** Rupal I. Mehta, Rajiv Mangla, Rashi I. Mehta

**Affiliations:** 1 Department of Pathology, Rush University Medical Center, Chicago, IL 60612, USA; 2 Rush Alzheimer’s Disease Center, Rush University Medical Center, Chicago, IL 60612, USA; 3 Department of Radiology, SUNY Upstate Medical University, Syracuse, NY 13210, USA; 4 Department of Neuroradiology, Rockefeller Neuroscience Institute, West Virginia University, Morgantown, WV 26506, USA; 5 Department of Neuroscience, Rockefeller Neuroscience Institute, West Virginia University, Morgantown, WV 26506, USA

**Keywords:** arachnoid granulation, dural lymphatic system, foam cell, giant arachnoid granulation, headache, LEC, lymphatic endothelium, lymphatic endothelial cell, macrophage, monocyte

## Abstract

Giant arachnoid granulations (GAGs) are poorly investigated. Here, we document clinical findings associated with five new GAGs and illustrate the anatomical composition of these structures as well as diagnostic considerations in three symptomatic adults. The GAGs ranged from 1.1 to 3.6 cm (mean, 2.2 cm) in maximum dimension and manifested in middle-aged individuals who presented with long-standing brain mass and/or chronic headache. On imaging examinations, the tissues appeared as irregular parasagittal and/or perisinus structures that demonstrated heterogeneous internal elements. The GAGs abutted dura, extended through calvarial marrow spaces, and impinged on dural venous sinuses, causing their stenosis. The histologic workup of two GAG specimens resected from separate individuals revealed central collagen with pronounced internal vascular proliferation. One specimen additionally exhibited reactive changes within the lesion, including venous thrombosis, hemorrhage, and conspicuous inflammation. The salient immune component consisted of a foam cell-rich infiltrate that obstructed subcapsular and internal sinusoidal GAG spaces. Within this specimen, meningothelial hyperplasia was also appreciated. Notably, proliferated lymphatic vascular elements were additionally observed within the structure, extending into deep central collagen regions and engulfing many extravasated erythrocytes in the subcapsular space. In both surgically treated patients, symptoms resolved completely following resection. This report is the first to definitively depict reactive vascular and immunological changes within GAGs that were clinically associated with headache. The frequency of reactive changes within these meningeal structures is unclear in the literature, as GAGs are rarely sampled and investigated. Further systematic analyses are warranted to elucidate the causes and consequences of GAG genesis and their roles in physiology and disease states.

## 1. Introduction

Arachnoid granulations (AG), also commonly known as Pacchionian bodies, are present along the meningeal surface of the mammalian brain and are primarily localized in parasagittal brain regions in the vicinity of dural venous sinuses (DVS) [1]. In humans, AGs typically measure only a few millimeters in diameter [1], but on occasion, they enlarge to form giant arachnoid granulations, or giant Pacchionian bodies (GAGs). When enlarged, these structures may be associated with clinical symptomatology [2,3,4,5,6,7]. GAGs have increasingly been reported in recent years, primarily in radiological literature [4,5,6,7], though their composition remains poorly established [1,2]. In our diagnostic practice, we prospectively encountered five new GAGs in middle-aged individuals who presented with scalp or calvarial masses and/or complaints of intermittent or chronic headache. The rarity of biopsy assessment of this tissue [1,2,3] as well as unexpected and symptomatic reactive changes in one patient prompted this report. Here, we document neuroimaging findings in five new GAGs that were diagnosed in three symptomatic adults and provide histopathological characterization that, for the first time, depicts prominent internal vascular components and reactive perivascular and immunological changes. These findings may aid diagnostic physicians who encounter symptomatic AGs or GAGs in live patients. Evidence provided here also suggests that these structures are dynamic tissues at brain borders and are susceptible to pathologies.

## 2. Case 1

### 2.1. Clinical Presentation

A patient presented with a complaint of chronic headaches for at least 30 years as well as a history of multiple syncopal episodes. The syncope was sometimes triggered by coughing. The headaches were bitemporal and characterized as a severe pressure sensation that was sometimes pulsating in nature, and occasionally continuous for up to a month in duration. Topiramate (Topamax^®^, Janssen Pharmaceuticals, Inc; Beerse, Belgium) was initiated several years prior to admission but had provided no relief.

### 2.2. Imaging Features 

**Figure 1:**
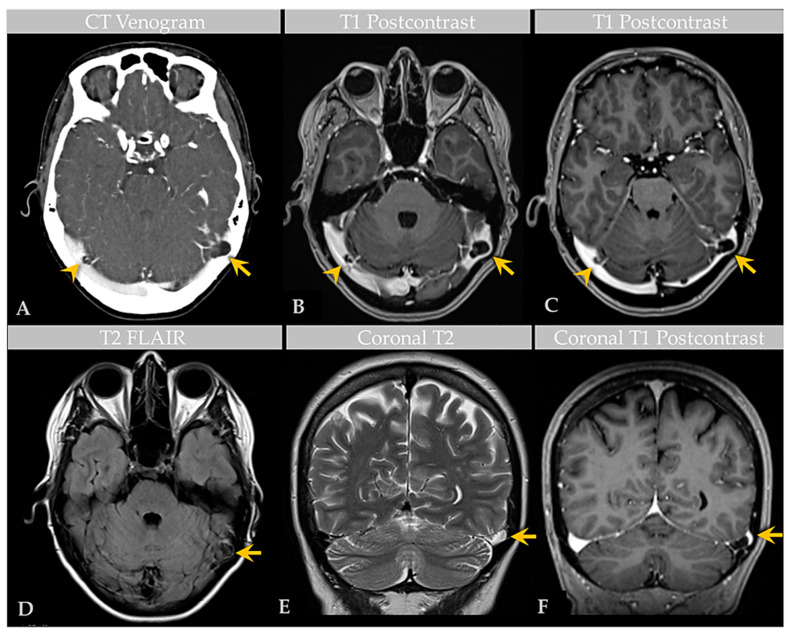
Case 1: Imaging of an individual with multiple GAGs. CT venogram (**A**) shows GAGs indenting the lateral aspect of bilateral TS (arrow, arrowhead). Postcontrast T1-weighted MRI images (**B**,**C**) demonstrate bilateral TS GAGs, measuring 1.6 cm on the left (arrow) and 1.1 cm on the right (arrowhead). The GAG is associated with focal severe narrowing of the lateral left TS (arrow, (**C**)). Axial T2-weighted FLAIR image (**D**) demonstrates heterogeneous signal within the GAG (arrow), likely reflecting internal soft tissue elements with CSF flow turbulence. Coronal T2-weighted (**E**) and postcontrast T1-weighted (**F**) images show high-grade TS luminal narrowing by the GAG (arrows). An additional SSS GAG is not depicted.

A recent MRI exam of the brain, performed at an outside institution, was interpreted as positive for bilateral transverse sinus thrombosis. The patient was transferred to our institution and underwent computed tomography (CT) venogram of the head (Figure 1). CT venogram analysis and reinterpretation of the brain MRI revealed bilateral GAGs that impinged on the lateral transverse sinuses (TS). The GAGs measured 1.1 cm on the right side and 1.6 cm on the left side and demonstrated a heterogeneous T2 signal (Figure 1). A third GAG that measured 1.5 cm and was sessile in morphology was also identified along the superior sagittal sinus (SSS). There was no TS or other DVS thrombosis.

### 2.3. Surgical Course

Evaluation for intracranial hypertension and potential DVS stenting was recommended.

### 2.4. General Histology Features

No biopsy specimen was obtained.

## 3. Case 2

### 3.1. Clinical Presentation

A patient was admitted for workup after presenting with a complaint of chronic headache. The patient described the headaches as migraines that had been intermittent over several years. Pain episodes began in the occipital region but often radiated toward the forehead. More recently, the patient began experiencing neck pain that radiated into the right shoulder, arm, and first three digits of the right hand. The patient also reported numbness and discomfort in these three digits as well as the radial aspect of the right hand, exacerbated by movement. Additionally, the patient described difficulty with right-sided head movement but denied noticing any masses or tenderness in the suboccipital region. Prior to presentation, the patient had undergone physical therapy, which led to minimal relief of symptoms. The family history was unremarkable, though the patient’s past medical history and review of systems were significant for balance disturbance, sinus headaches, hypertension, and mild memory problems. Physical examination of the head revealed a firm, non-tender, palpable mass in the suboccipital region. A neck exam revealed discomfort and reduced range of motion to the right side. Motor examination revealed minimal weakness in the right brachioradialis and right upper digits, upon digital extension. A sensory exam revealed diminished sensation in the right C6 distribution. Neurological examination was otherwise unremarkable. 

### 3.2. Imaging Features

**Figure 2:**
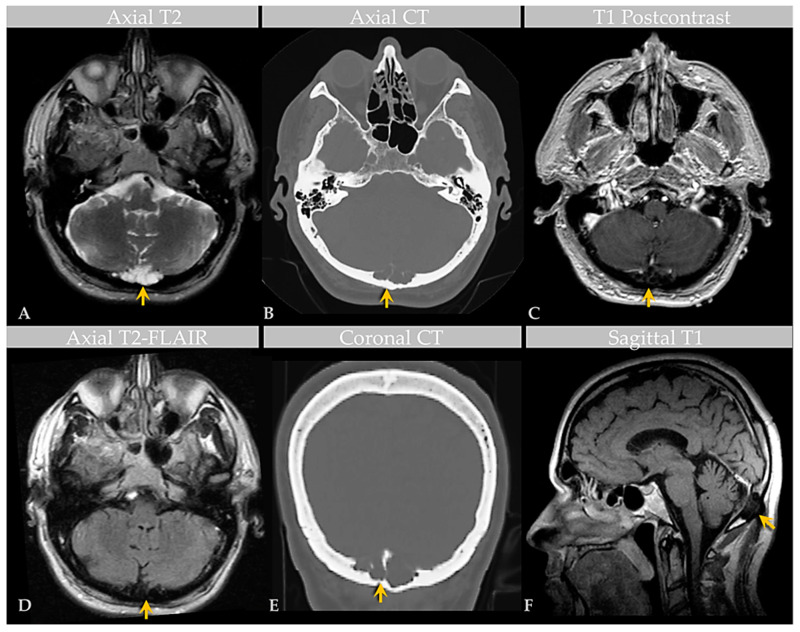
Case 2: Imaging of an individual with GAGs involving the confluence of sinuses. MRI (**A**) and CT (**B**) images show a GAG measuring over 3.6 cm centered in the region of the torcula horophili eroding through the diploic space and remodeling the outer cortex of the occipital bone (arrows). Note that the diploic space is expanded. There are thin, nonenhancing septae within the AG, as shown on postcontrast T1-weighted (**C**) and T2-FLAIR (**D**) MRI images, causing internal signal heterogeneity. Features are also demonstrated in the coronal CT image (**E**) and sagittal T1-weighted image (**F**). Images are from the same individual depicted in Figure 3.

**Figure 3:**
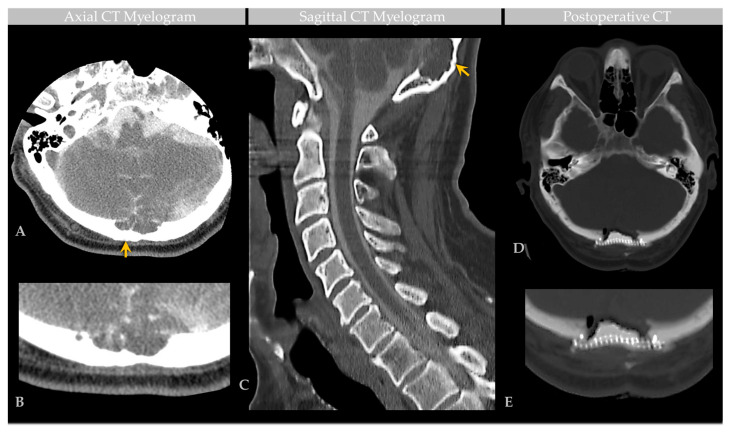
Case 2: CT Myelogram of an individual with GAG involving the confluence of sinuses. Axial sections of CT myelogram, with zoomed image, show contrast partly filling the GAG, as shown on two contiguous levels (**A**,**B**). Sagittal image (**C**) shows soft tissue attenuation within the GAG with defect of the inner table, diploic expansion, and endosteal scalloping of the outer calvarial cortex (arrow). Postoperative CT (**D**) demonstrates occipital cranioplasty with bone cement and metallic mesh following lesion resection, as shown to advantage on zoomed section (**E**). Images are from the same individual depicted in Figure 2.

Brain MRI with and without contrast and noncontrast CT of the head (Figure 2) revealed a non-enhancing 3.6 × 1.3 × 1.8 cm abnormality that eroded the inner table of the occipital bone, causing lytic marrow expansion, extension through the dura and to the inion and the level of the torcula. Heterogeneous internal cystic-appearing MRI signal characteristics raised the possibility of an epidermoid cyst. There was no enhancing mass. MRI of the cervical spine did not reveal any abnormality to explain the patient’s symptomatology, though diffuse degenerative disease was appreciated. No definite cause for the radiculopathy was identified. A prior CT study documented the absence of the observed cranial lesion seven years prior to admission. To further evaluate the spinal cord, subarachnoid space, and associated structures, a CT myelogram was also performed (Figure 3) and revealed partial penetration of iopamidol (Isovue-M^®^ 300) contrast tracer into the GAG interior, which demonstrated prominent internal soft tissue elements.

### 3.3. Surgical Course

Due to the persistent symptoms and unusual imaging findings, suboccipital craniectomy and surgical excision of the mass were performed and sent for biopsy workup. An intraoperative biopsy of 0.6 to 0.8 cm fragments of fresh tissue returned a diagnosis of benign bone. Titanium mesh cranioplasty was performed. The patient reported significant improvement in the headaches immediately following surgery.

### 3.4. General Histology Features

A larger resected mass measuring 3.2 × 3.0 × 1.0 cm was received in the pathology lab for permanent section analysis. The specimen was decalcified and then underwent routine processing. On histology, the biopsy material revealed fragments of benign bone and collagen tissue with a proliferation of small blood vessels. The mass was initially interpreted as a cavernous hemangioma, but on subsequent evaluation and radiological–pathological correlation, it was reinterpreted as a GAG. The associated bone fragments exhibited evidence of remodeling and reactive change without evidence of malignancy or other pathological changes. The patient reported complete resolution of the headaches following GAG resection.

## 4. Case 3

### 4.1. Clinical Presentation

A patient reported a new onset of unremitting headache and irritation of a chronic scalp “cyst” following an episode of mild closed head injury. The patient had a long-standing history of two presumptively diagnosed GAGs that were present at the skull vertex. At presentation, a large left parietal bone mass was readily palpable on physical examination with intact overlying skin. No meningeal signs or focal neurological deficits were exhibited. Due to the unclear etiology of the symptoms, the patient was admitted for workup of the larger calvarial mass that was associated with the patient’s headaches. 

### 4.2. Imaging Features 

**Figure 4:**
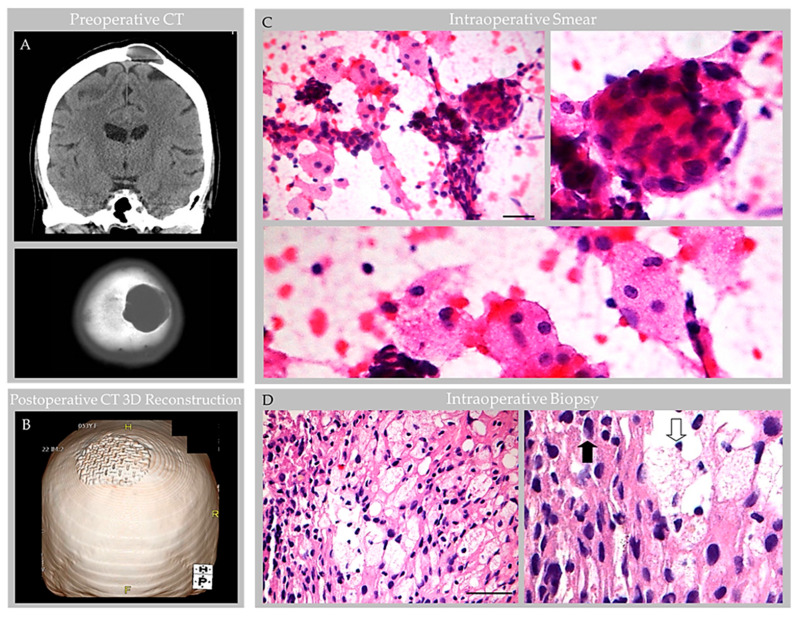
Case 3: CT and intraoperative histology images in an individual with parietal GAG. Preoperative noncontrast head CT images at the level of the parietal vertex (**A**) demonstrate a left calvarial lytic mass with smooth, sclerotic margins causing diploic expansion and endosteal scalloping with internal soft tissue and fluid attenuation, as shown on coronal (top) and axial (bottom) images. Postoperative CT three-dimensional (3D) reconstruction (**B**) demonstrates craniopasty with mesh reconstruction at the GAG resection site. During intraoperative consultation, smear (**C**) and frozen section (**D**) preparations of biopsy material revealed heterogeneous elements. On smear (**C**), meningothelial tissue was observed in tight whorls (inset, upper-right-hand image) and foam cells were scattered singly and arranged in loose sheets (inset, bottom image). On frozen section (**D**), meningothelial cells with nuclear pseudoinclusions (inset, closed arrow) and foam cells (inset, open arrow) were further visualized. Scale bars: (**C**), 20 µm; (**D**), 50 µm. Images are from the same patient depicted in Figure 5, Figure 6, Figure 7 and Figure 8.

Noncontrast head CT revealed cystic diploic expansion of the left parietal calvarium with an associated inner cortical defect, outer cortical endosteal scalloping, and bony remodeling (Figure 4A,B). The lesion demonstrated central hypodensity with peripheral soft tissue attenuation. On MRI, it measured 3.4 × 3.4 × 1.3 cm and demonstrated internal cerebrospinal fluid (CSF) signal with a thin peripheral rim of restricted diffusion and communication with the subarachnoid space. No enhancing mass, edema, or evidence of hemorrhage was seen. The lesion abutted the SSS, which was patent on the MR venogram. Imaging supported a nonaggressive lesion such as a GAG or benign bone cyst.

### 4.3. Surgical Course

At surgery, a circumscribed mass was visualized within thinned calvarial bone following the retraction of scalp skin and periosteum. After outer cortical bone resection, the mass apex was dissected from adjacent bone and an intraoperative biopsy was obtained for smear and frozen section analysis. Hematoxylin-and-eosin (H&E)-stained preparations of the biopsy material revealed bland meningothelial cells arranged in sheets and whorls, admixed with foam cells (Figure 4C,D). Intraoperative pathology consultation further confirmed the presence of a fluctuant meningeal-based lesion that originated from arachnoid mater and extended into adjacent dura and bone tissue. The structure was composed of central collagen and with gentle manipulation, CSF was expressed from its interior. Following partial resection of the dome, the structure was partially drained, a ligature was placed around the lesion base and duraplasty and titanium mesh cranioplasty were performed. The patient’s headache improved following surgery and postoperative imaging showed expected postsurgical changes. The patient was discharged and on follow-up reported resolution of the headaches.

### 4.4. General Histology Features

**Figure 5:**
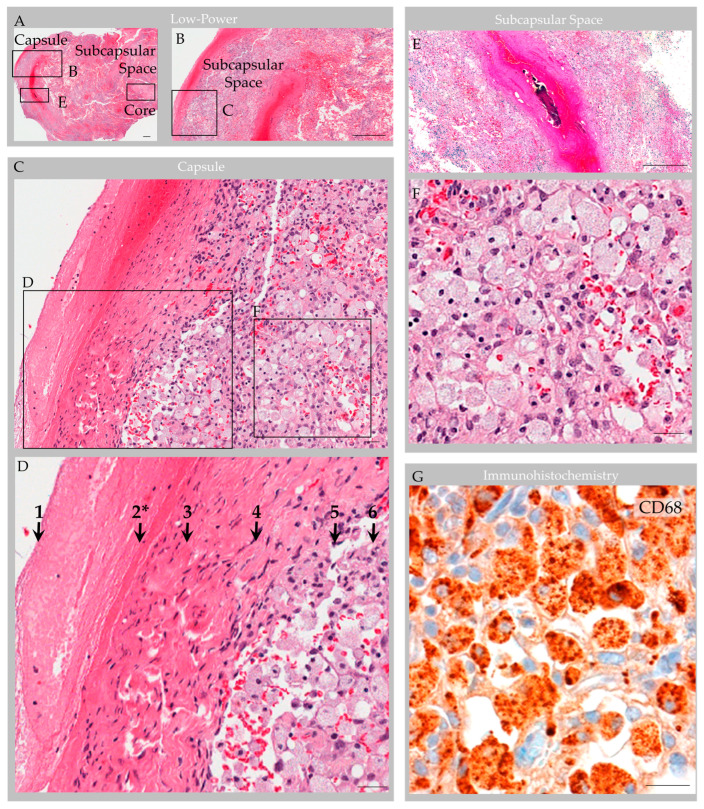
Case 3: Histology images, capsule and subcapsular regions in an individual with parietal GAG. Hematoxylin and eosin-stained sections revealed a polypoid structure composed of capsule, subcapsular space, and central collagen core tissue (**A**). The capsular elements (**B**–**D**) consisted of fibrin (1); endothelium (2 *); dense collagen, i.e., dura (3); and arachnoid cells (4), which covered a subcapsular space (5) that contained foam cells (6). A thrombosed vein with early organization and focal calcification, congestion and associated hemorrhage was also observed in subcapsular space (**E**) with adjacent foam cells (**F**). Immunohistochemistry highlighted CD68-positive macrophages within the subcapsular space (**G**), and these cells extended deeper into the GAG tissue.. Scale bars: (**A**,**B**,**E**), 100 µm; (**C**,**D**,**F**,**G**) 10 µm. * Arrow “2” indicates the region of vascular endothelial lining, which was present focally on deep sections. Images are from the same individual depicted in Figure 4, Figure 6, Figure 7 and Figure 8.

**Figure 6:**
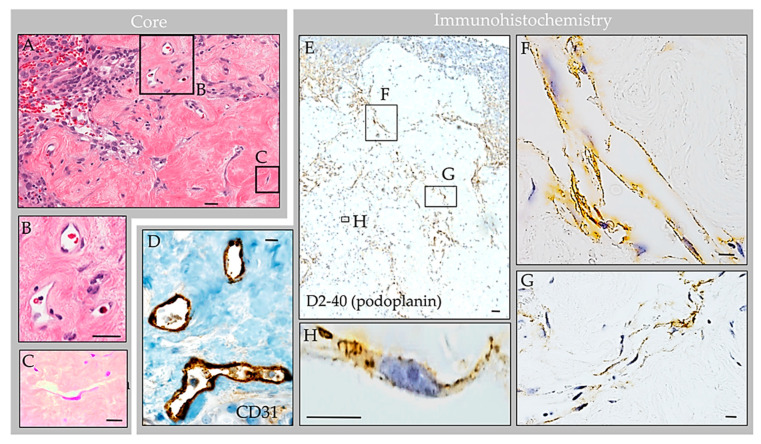
Case 3: Histology images, central collagen core region in an individual with parietal GAG. H&E-stained sections of the core revealed dense collagen core tissue (**A**) with embedded vascular structures (**B**), fibroblasts, foam cells, and thin flattened cells (**C**). Immunohistochemistry highlighted CD68-positive macrophages within internal sinuses, CD31-positive erythrocyte-containing vessels within the collagen core (**D**) and D2-40 (podoplanin)-positive erythrocyte-associated and erythrocyte-unassociated LECs within the subcapsular space and collagen coI (**E**). The LECs were arranged in well-formed tubular lymphatic vessels (**F**); non-tubularized linear aggregates (**G**); and singly scattered cells (**H**). Scale bars: (**A**,**E**), 20 µm; (**B**–**D**,**F**–**H**), 5 µm. Images are from the same individual depicted in Figure 4, Figure 5, Figure 7 and Figure 8.

Routine processing and H&E staining of the 2.3 × 1.3 × 0.3 cm resection material revealed fragments of unremarkable bone and periosteum. The associated soft tissue mass consisted of an encapsulated, polypoid tissue (Figure 5A,B). Its capsular surface displayed dura with adherent fibrin and focal endothelial lining with subjacent meningothelium (Figure 5C,D) [1]. Beneath this multilaminar capsule was an expanded subcapsular space and deep collagen tissue (Figure 5A). The subcapsular space contained an organized thrombosed vein with focal calcification (Figure 5E). Also present was moderate hemorrhage and abundant foam cells (i.e., reactive monocytes and macrophages) that obliterated the subcapsular space (Figure 5F,G) and infiltrated into sinuses of the central collagen core region (Figure 6A) [1]. Notably, erythrocyte-containing and erythrocyte-devoid vascular channels were present in the subcapsular space and within the collagen core (Figure 6A,B), along with scattered fibroblasts and clusters of thin, flattened cells (Figure 6C). Scant plasma cells and lymphocytes were observed within the structure, but no tumor or other abnormalities were detected.

### 4.5. Immunohistochemistry Features 

**Figure 7:**
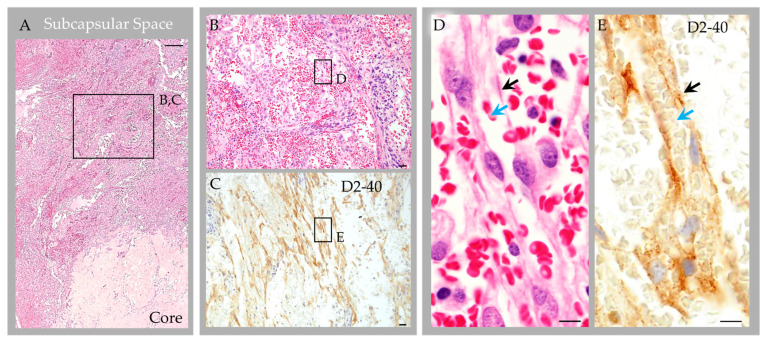
Case 3: Histology images, deep subcapsular space region in an individual with parietal GAGs. Hematoxylin and eosin-stained sections revealed abundant extravasated erythrocytes in the deep subcapsular space region, adjacent to the core (**A**), as shown in an enlarged cropped image (**B**). The extravasated erythrocytes intermingle with LEC, which were abundantly present and were arranged in a prominent linear pattern, as highlighted by D2-40 immunostain (**C**). On higher magnification images (**D**,**E**) representing boxed areas in (**B**) and (**C**), respectively, LEC and poorly formed lymphatic vascular channels (black arrows) are seen surrounding and engulfing extravasated erythrocytes (blue arrows). Scale bars: (**A**), 100 µm; (**B**,**C**), 20 µm; (**D**,**E**), 5 µm. Images are from the same individual depicted in Figure 4, Figure 5, Figure 6 and Figure 8.

Labeling with anti-epithelial membrane antigen (EMA) antibody highlighted meningothelial cells within the capsule and core [1]. Labeling with anti-CD31 and CD34 antibodies highlighted internal blood vessels next to and embedded within the collagen core (Figure 6D). There was also focal label in superficial capsular endothelium that was present in a focal region only [1]. Labeling with anti-CD68 antibodies confirmed the presence of abundant foamy monocytes and/or macrophages throughout the expanded subcapsular space and intertrabecular sinuses of the collagen core (Figure 5G). A separate subset of vessels and cells present in the subcapsular space and central core were also labeled strongly and diffusely with anti-D2-40 (podoplanin) antibody (Figure 6E). The D2-40-expressing cells were arranged in central collagen as well-formed, organized vascular tubules that were devoid of erythrocytes (Figure 6F), consistent with typical lymphatic vessels; aggregates in non-tubularized linear pattern (Figure 6G); and, in deeper regions, as singly scattered lymphatic endothelial cells (LECs) (Figure 6H). The D2-40 expressing cells in the subcapsular space region were arranged in a similar pattern. However, in this region, where extravasted erythrocytes were frequent, the LECs and lymphatic vessels appeared to engulf and contain many extravasated red blood cells (Figure 7).

**Figure 8:**
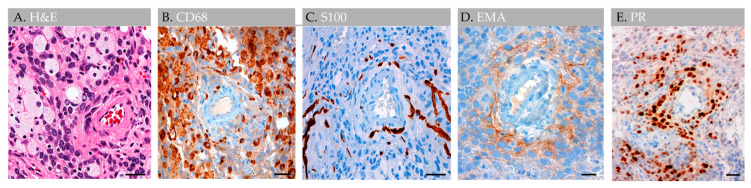
Case 3: Histology images, perivascular region, in an individual with parietal GAGs. H&E-stained section of an internal GAG vessel reveals perivascular cell aggregates (**A**), including cuffing by CD68-positive foam cells (**B**), S100-positive nerve twigs (**C**), and EMA-positive (**D**) and progesterone receptor-positive (**E**) meningothelial cells, suggesting perivascular molecular exchange and signaling in this region. Abbreviation: EMA, epithelial membrane antigen; PR, progesterone receptor. Scale bars: (**A**–**E**), 20 µm. Images are from the same individual depicted in Figure 4, Figure 5, Figure 6 and Figure 7.

Throughout the lesion, anti-CD1a, pan-keratin, inhibin, kappa, lambda, and amyloid (4G8) antibody labels were all negative, whereas anti-Ki67, CD138, CD45, and progesterone receptor (PR) labels highlighted scant cells only (i.e., less than 0.5% of total cells); S100 highlighted rare nerve twigs in perivascular regions but was negative in the remaining lesion [1]. Notably, EMA- and PR-positive meningothelial cells were also present in perivascular regions (Figure 8).

### 4.6. Special Staining Features 

An extensive panel of special stains including Gram, Grocott’s methenamine silver, Periodic acid–Schiff, and acid-fast bacillus, each performed with deep section repeats on each of two blocks, was additionally negative for microorganisms, and Congo Red stain further confirmed the absence of any amyloid material. 

## 5. Discussion

GAGs are well documented in radiological literature [4,5,6,7] and have been reported in various locations across human brain surfaces [2,3,4,5,6,7]. However, their prevalence and etiologies are minimally investigated and evidence concerning GAG internal microstructure is limited. Despite scant anatomical evidence, the potential for GAGs to cause clinical symptomatology is well known [3,4,5,6,7]. Moreover, a recent investigation of non-giant AG anatomy [1] revises concepts on the AG structure and function in humans [8,9,10]. This recently published work systematically analyzed AG histology in persons across age and documented the presence of vessels and various immune cells within AG interiors, highlighting neuroimmune roles of these meningeal bodies that likely surveil CNS interfaces [1]. However, this prior work incorporated postmortem tissues from non-diseased brains, only. Here, we studied available anatomical data associated with five GAGs from three individuals who presented with neurological symptoms to better understand the GAG structure based on in vivo and ex vivo features. On imaging examinations, GAGs indented bone and/or DVS and varied in size from 1.1 cm to 3.6 cm (mean, 2.2 cm). We also discovered that two of two (100%) GAGs with available H&E-labeled biopsy evidence showed florid internal vascular proliferation. One of the two biopsied GAGs (50%) also showed evidence of heterogeneous elements with exuberant inflammation. Additional internal reactive changes, including venous thrombosis and hemorrhage with early thrombus organization and calcification, were present in this latter GAG specimen that was sampled from an individual who sustained recent head trauma. The immune cell infiltrate in this patient displayed mixed constituents but was primarily comprised of foam cells that permeated and obliterated the subcapsular and central sinusoidal spaces. Meningothelial hyperplasia and associated nuclear PR expression was also focally present in this specimen and was most prominent in perivascular locations, suggesting important perivascular molecular exchange and signaling in AG and GAG tissues.

Given the prominent and heterogenous nature of the reactive changes identified within this latter specimen (i.e., Case 3), the resection material was further characterized with a comprehensive immunohistochemistry panel. Importantly, labeling with anti-CD31, CD34 and D2-40 antibodies and counterstain revealed that internal vessels within the structure consisted of erythrocyte-containing and erythrocyte-devoid blood and lymphatic vascular channels. D2-40-labeled lymphatic vascular elements within the GAGs consisted of well-formed tubularized lymphatic vessels as well as loosely formed lymphatic channels and singly scattered LECs that haphazardly extended into spaces of the central collagen core [1], appearing to have a proliferative edge. The lymphatic vessels and LEC encircled and engulfed extravasated erythrocytes in the subcapsular space. To our knowledge, the presence of lymphatic vessels, lymphatic elements and LECs within central GAG tissue has not previously been reported in the literature and this feature was not identified in non-giant AGs that were recently thoroughly investigated from frontal brain regions of asymptomatic persons of variable age [1]. Given this novel finding in a reactive GAG from an otherwise healthy middle-aged adult, in combination with the observation that the patient’s headaches resolved following partial GAG dome resection and drainage, we suspect that the obstructive hemorrhage, immune cell infiltrate, and associated flow stasis within the GAG structure were causative of the patient’s headaches [7].

In light of historical literature and generally accepted knowledge regarding human AG structure [8,9,10], the diagnosis of GAGs may be challenging in some patients. Detailed radiologic–pathologic data pertaining to GAGs are not available in published literature, although internal vascular components are well described in radiological reports [4,5,6,7]. Diagnostic challenges are exemplified by the differential diagnoses and initial diagnostic impressions rendered in the cases presented here. The first case was originally interpreted on imaging studies as bilateral transverse sinus thrombosis, which delayed proper diagnostic workup and management in this patient who experienced persistent headaches over the course of decades and was ultimately determined to have multiple GAGs involving three distinct brain regions. Likewise, the second case was interpreted on imaging studies as a possible GAG versus epidermoid cyst, and on biopsy was interpreted initially as a cavernous hemangioma, but consultation and radiologic–pathologic evaluation ultimately confirmed the diagnosis of GAGs in this patient. The third case was suspected to be a GAG based on characteristic imaging features and surgical inspection, and the diagnosis was confirmed by radiological–pathological correlation following biopsy. However, imaging features also raised the possibility of a benign bone cyst while the histology features in some areas closely mimicked a meningioma, cavernous hemangioma, and/or infection given the focal arrangement and hyperplasia of meningothelium, collagen and vessels in combination with the accumulation of immune cells. Follow-up in two patients (i.e., case two and case three), performed 2 months to 4 years after surgery, was notable for the absence of headache and MRI recurrence. 

As with asymptomatic non-giant AG, investigation of GAG tissue is challenging in live humans [1]. A recent study demonstrated that internal collagen forms a stromal meshwork within AGs and depicted, for the first time, the presence of cytokine and immune cell enrichment within AG cores [1], which has revised concepts pertaining to AG biology as these findings suggested that AGs have underrecognized implications in neurophysiology. This prior study also demonstrated that AGs increase in size and have altered composition across the lifespan, raising the hypothesis that chronic flow stasis and uncharacterized degenerative changes may be involved in their reorganization and/or enlargement. While GAGs are rarely biopsied from live persons, the present report provides new data regarding their possible internal composite. Here, we show the potential for prominent reactive vascular and immune changes within enlarged AGs. These features within a prominently enlarged specimen further support that AGs and GAGs likely have important roles in vascular and immune homeostatic mechanisms. Importantly, the ingrowth of lymphatic vascular elements shown here for the first time supports the role of AGs in neurofluid, antigen and macromolecular processing. Additional preclinical and clinical studies are warranted to further elucidate GAG structure and function in health and aging as well as across various disease states.

## Data Availability

Not applicable.

## Abbreviations

AG	arachnoid granulation
CSF	cerebrospinal fluid
CT	computed tomography
DVS	dural venous sinus
EMA	epithelial membrane antigen
FLAIR	fluid attenuated inversion recovery
GAG	giant arachnoid granulation
H&E	hematoxylin-and-eosin
LEC	lymphatic endothelial cell
MRI	magnetic resonance imaging
PR	progesterone receptor
SSS	superior sagittal sinus
TS	transverse sinus

## References

[1] Shah T., Leurgans S.E., Mehta R.I., Yang J., Galloway C.A., de Mesy Bentley K.L., Schneider J.A., Mehta R.I. (2023). Arachnoid granulations are lymphatic conduits that communicate with bone marrow and dura-arachnoid stroma. J. Exp. Med..

[2] Haybaeck J., Silye R., Soffer D. (2008). Dural arachnoid granulations and “giant” arachnoid granulations. Surg. Radiol. Anat..

[3] Rosenberg A.E., O’Connell J.X., Ojemann R.G., Plata M.J., Palmer W.E. (1993). Giant cystic arachnoid granulations: A rare cause of lytic skull lesions. Hum. Pathol..

[4] Chin S.C., Chen C.Y., Lee C.C., Chen F.H., Lee K.W., Hsiao H.S., Zimmerman R.A. (1998). Giant arachnoid granulation mimicking dural sinus thrombosis in a boy with headache: MRI. Neuroradiology.

[5] De Keyzer B., Bamps S., Van Calenbergh F., Demaerel P., Wilms G. (2014). Giant arachnoid granulations mimicking pathology. A report of three cases. Neuroradiol. J..

[6] Kiroglu Y., Yaqci B., Cirak B., Karabulut N. (2008). Giant arachnoid granulation in a patient with benign intracranial hypertension. Eur. Radiol..

[7] Peters S.A., Frombach E., Heyer C.M. (2007). Giant arachnoid granulation: Differential diagnosis of acute headache. Australas. Radiol..

[8] Netter F.H. (2019). Atlas of Human Anatomy.

[9] Upton M.L., Weller R.O. (1985). The morphology of cerebrospinal fluid drainage pathways in human arachnoid granulations. J. Neurosurg..

[10] Kida S., Yamashima T., Kubota T., Ito H., Yamamoto S. (1988). A light and electron microscopic and immunohistochemical study of human arachnoid villi. J. Neurosurg..

